# The histone chaperone Spt6 is required for normal recruitment of the capping enzyme Abd1 to transcribed regions

**DOI:** 10.1016/j.jbc.2021.101205

**Published:** 2021-09-17

**Authors:** Rajaraman Gopalakrishnan, Fred Winston

**Affiliations:** Department of Genetics, Blavatnik Institute, Harvard Medical School, Boston, Massachusetts, USA

**Keywords:** *Saccharomyces cerevisiae*, transcription, Spt6, Abd1, mRNA capping, ChIP, chromatin immunoprecipitation, FA, formaldehyde, FDR, false discovery rate, IgG, immunoglobulin G, IP, immunoprecipitation, MS, mass spectrometry, PAF, polymerase-associated factor, RNAPII, RNA polymerase II, TF, transcription elongation factor, TMT, tandem mass tag, YPD, yeast extract–peptone–dextrose

## Abstract

The histone chaperone Spt6 is involved in promoting elongation of RNA polymerase II (RNAPII), maintaining chromatin structure, regulating cotranscriptional histone modifications, and controlling mRNA processing. These diverse functions of Spt6 are partly mediated through its interactions with RNAPII and other factors in the transcription elongation complex. In this study, we used mass spectrometry to characterize the differences in RNAPII-interacting factors between wildtype cells and those depleted for Spt6, leading to the identification of proteins that depend on Spt6 for their interaction with RNAPII. The altered association of some of these factors could be attributed to changes in steady-state protein levels. However, Abd1, the mRNA cap methyltransferase, had decreased association with RNAPII after Spt6 depletion despite unchanged Abd1 protein levels, showing a requirement for Spt6 in mediating the Abd1–RNAPII interaction. Genome-wide studies showed that Spt6 is required for maintaining the level of Abd1 over transcribed regions, as well as the level of Spt5, another protein known to recruit Abd1 to chromatin. Abd1 levels were particularly decreased at the 5′ ends of genes after Spt6 depletion, suggesting a greater need for Spt6 in Abd1 recruitment over these regions. Together, our results show that Spt6 is important in regulating the composition of the transcription elongation complex and reveal a previously unknown function for Spt6 in the recruitment of Abd1.

During transcription elongation, RNA polymerase II (RNAPII) interacts with a large set of proteins. These include proteins that promote processivity of RNAPII such as transcription elongation factor (TF) IIS and the 5,6-dichloro-1-β-d-ribofuranosylbenzimidazole sensitivity–inducing factor complex (composed of Spt4/5), histone chaperones such as Spt6 and FACT (facilitates chromatin transcription), histone modification enzymes such as Set2, and enzymes involved in cotranscriptional processes such as mRNA capping and splicing (1, 2). The concerted action of these factors ensures efficient transcription by RNAPII and mRNA processing, producing a mature mRNA molecule that can be exported to the cytoplasm for translation. For many of these TFs, our understanding of how they regulate transcription is limited.

The histone chaperone Spt6 is a highly conserved and multifunctional protein that is essential for viability in *Saccharomyces cerevisiae* (3, 4) and metazoans (5, 6, 7). Spt6 directly interacts with RNAPII through its C-terminal tandem SH2 domains, and it is part of the transcription elongation complex (8, 9, 10, 11, 12, 13, 14). The localization of Spt6 over transcribed regions positively correlates with the level of RNAPII, suggesting an important role for Spt6 in transcription elongation (15, 16), and there is evidence that Spt6 is required for normal elongation *in vivo* and *in vitro* (17, 18). Spt6 also interacts with all four histones (19, 20), and it is required for chromatin organization over transcribed regions (21, 22, 23, 24). Strikingly, in *spt6* mutants, there is a genome-wide increase in the expression of transcripts originating from within gene bodies in both sense and antisense orientations (21, 22, 25, 26, 27). Together, these results have established that Spt6 is required for maintaining chromatin structure and transcriptional fidelity.

Spt6 regulates transcription elongation and cotranscriptional processes partly through its interactions with other proteins in the transcription elongation complex. Spt6 physically interacts with Spt5, and both proteins are required for normal transcription elongation (14, 18, 28, 29). Spt6 also recruits the polymerase-associated factor (PAF) complex (30) to transcribed regions (17, 23, 31) and can stimulate the ability of this complex to promote transcription elongation (14). The N-terminal region of Spt6 interacts with Spn1/Iws1 (32, 33). The Spt6–Spn1 interaction is necessary for maintaining H3K36 methylation, which is cotranscriptionally deposited in yeast and human cells (34, 35, 36). In yeast, Spt6 is required for promoting the activity of the H3K36 methyltransferase Set2 (37, 38, 39). In humans, the Spt6–Spn1 interaction also promotes mRNA splicing and export (40). Given the wide range of functions of Spt6, it presents an ideal system to understand the link between transcription elongation and the regulation of cotranscriptional processes.

In this study, we set out to characterize the role of Spt6 in the transcription elongation complex using *spt6*-*1004*, a widely used temperature-sensitive allele of *SPT6* (22, 25). After a shift to the nonpermissive temperature, *spt6-1004* cells become depleted for Spt6 protein (22) and show genome-wide reduction of nucleosome positioning and occupancy (15, 21, 22), loss of H3K36 methylation (37, 38), and expression of intragenic transcripts (21, 22, 25, 26, 27). To comprehensively characterize the set of proteins whose interaction with RNAPII is Spt6 dependent, we compared the RNAPII-interacting proteins between wildtype and *spt6-1004* cells. We identified 58 proteins that have altered association with RNAPII in *spt6-1004*, including decreased association of Spt5, Spn1, and PAF complex subunits, indicating a central role for Spt6 in regulating the composition of the transcription elongation complex. Interestingly, we find that the interaction of the mRNA cap methyltransferase Abd1 with RNAPII is decreased in *spt6-1004*. However, the other two capping enzymes, Cet1 and Ceg1, have unaltered interaction with RNAPII, suggesting a role for Spt6 in specifically mediating the interaction of Abd1 with the RNAPII elongation complex. Chromatin immunoprecipitation (IP) (ChIP)-Seq analysis shows a genome-wide decrease in Abd1, Spt5, and RNAPII levels on chromatin in *spt6-1004*. The decrease in Abd1 occupancy is particularly apparent at the 5′ ends of genes, which might be due to reduced Spt5 binding at the same region. In summary, our results provide new insights into the role of Spt6 in mediating interactions of other factors with RNAPII during transcription elongation.

## Results

### Purification of RNAPII complexes from wildtype and *spt6-1004* cells

**Figure 1:**
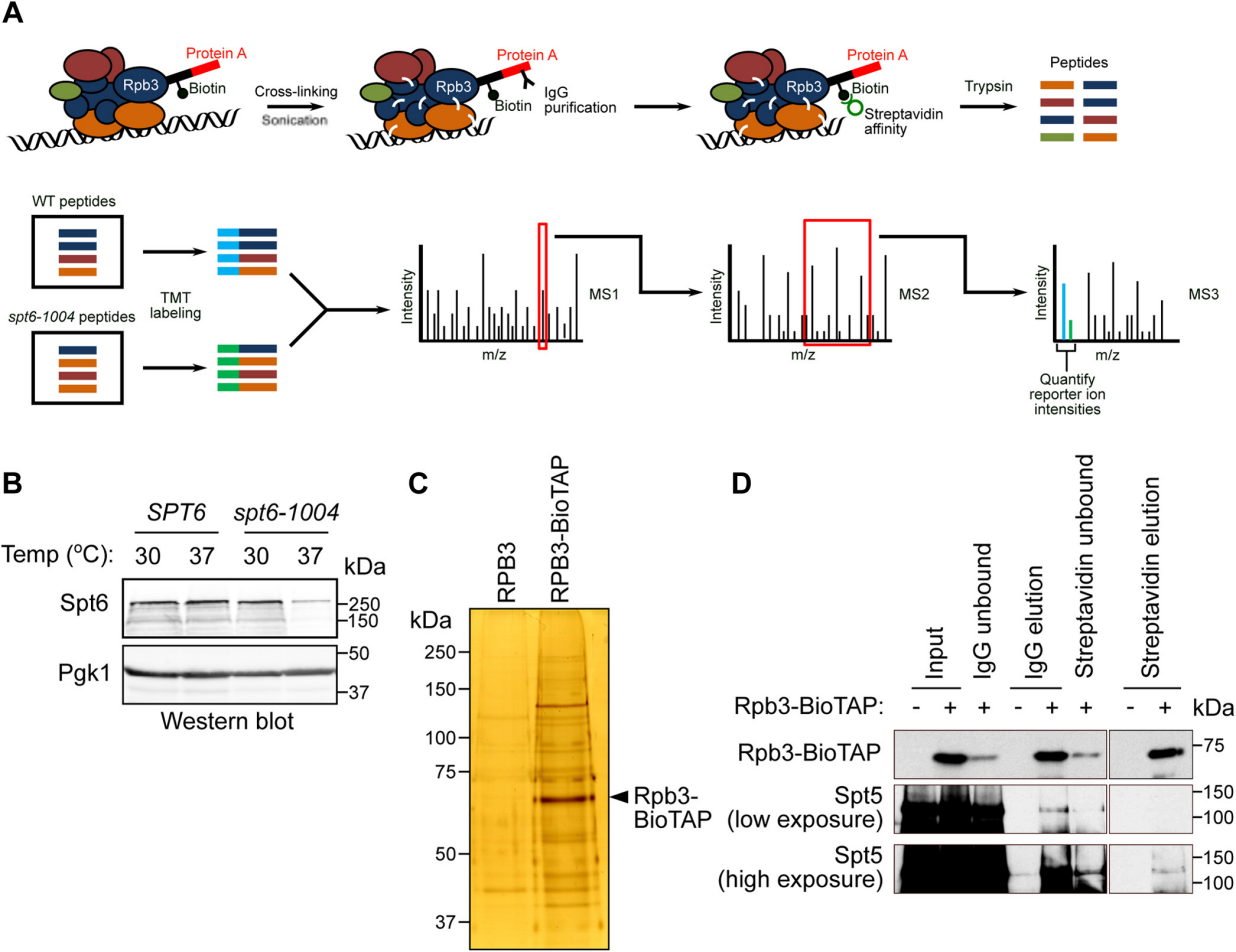
**Purification of RNAPII complexes by BioTAP-XL.***A*, schematic showing the BioTAP-XL procedure followed by MS. The cross-linked protein complexes are sonicated to solubilize chromatin-bound proteins. The protein complexes are purified using a two-step process—first using immunoglobulin G that binds to the protein A portion of the tag on Rpb3, and then using streptavidin that binds to the biotinylated portion of the tag. The purified complexes are subjected to on-bead trypsin digestion. The eluted peptides from the different samples are labeled with isobaric tandem mass tags and analyzed by triple-stage MS. The peptide abundances are obtained by quantifying the reporter ion intensities for each sample at the MS3 stage. *B*, Western blot showing Spt6 protein levels in wildtype and *spt6-1004* cells before (30 °C) and after a temperature shift to 37 °C. Pgk1 served as a loading control. *C*, representative silver-stained gel showing purification of Rpb3 from *spt6-1004* cells with and without the BioTAP tag on Rpb3. *D*, Western blot showing the abundance of Rpb3 and Spt5 at the different stages of purification. RNAPII, RNA polymerase II.

To identify factors whose interaction with RNAPII is dependent on Spt6, we purified RNAPII complexes from wildtype and *spt6-1004* cells using BioTAP-XL—a two-step purification method originally developed for *Drosophila* cells (41) and adapted here for *S. cerevisiae* (Fig. 1*A*). To do this, we fused the C-terminal end of Rpb3 (a subunit of RNAPII) to a tandem affinity tag consisting of protein A and a protein sequence that can be efficiently biotinylated *in vivo*. Wildtype and *spt6-1004* cells expressing the tagged *RPB3* gene were grown at 30 °C and shifted to the nonpermissive temperature (37 °C) for 80 min prior to formaldehyde (FA) crosslinking and cell harvesting. This temperature shift leads to depletion of Spt6 protein in *spt6-1004* cells (Fig. 1*B*) and exacerbation of mutant phenotypes without impairing the viability of the cells (22). Following crosslinking of protein complexes and cell lysis, a two-step purification of Rpb3 was performed, first using immunoglobulin G (IgG), which binds to protein A, and second using streptavidin that binds to biotin (Fig. 1*A*). A representative silver-stained gel showed that the purification of Rpb3 from *spt6-1004* cells was dependent upon the tag (Fig. 1*C*). As a positive control for the purification of RNAPII-associated factors, we tested for the copurification of Spt5 with Rpb3. Western blots showed that Spt5 copurified with Rpb3 only in purifications done from Rpb3-tagged cells (Fig. 1*D*).

### Identification and comparison of RNAPII-interacting proteins in wildtype and *spt6-1004* cells

To identify the proteins associated with RNAPII in wildtype and *spt6-1004* cells, we analyzed purified Rpb3 complexes by mass spectrometry (MS). As a control for specificity for association with RNAPII, we also analyzed proteins purified from untagged wildtype and *spt6-1004* cells. All samples were prepared and analyzed in duplicate, starting from two independent yeast colonies (biological replicates). Peptides from each sample were labeled with tandem mass tags (TMTs) to permit multiplexing and quantitative comparisons of protein levels between samples (42). The reporter ion intensities for each peptide were quantified at the MS3 stage (Fig. 1*A*), which provides greater accuracy because of lower contamination from other peptides having a similar *m/z* ratio as compared with quantification at the MS2 stage (43). The peptide intensities were normalized to the total peptide intensities within each sample. Averaged peptide intensities for each protein correlated well between the two replicates for each genotype (Pearson *R* between 0.93 and 0.99) (Fig. S1*A*). Upon comparing normalized protein abundances of the 12 RNAPII subunits between Rpb3-tagged and Rpb3-untagged samples, we observed higher abundances for all 12 RNAPII subunits in the Rpb3-tagged samples compared with the untagged samples (Fig. S1*B*). This verified that our normalization technique preserved the enrichment of Rpb3-specific interactors in the tagged samples.

**Figure 2:**
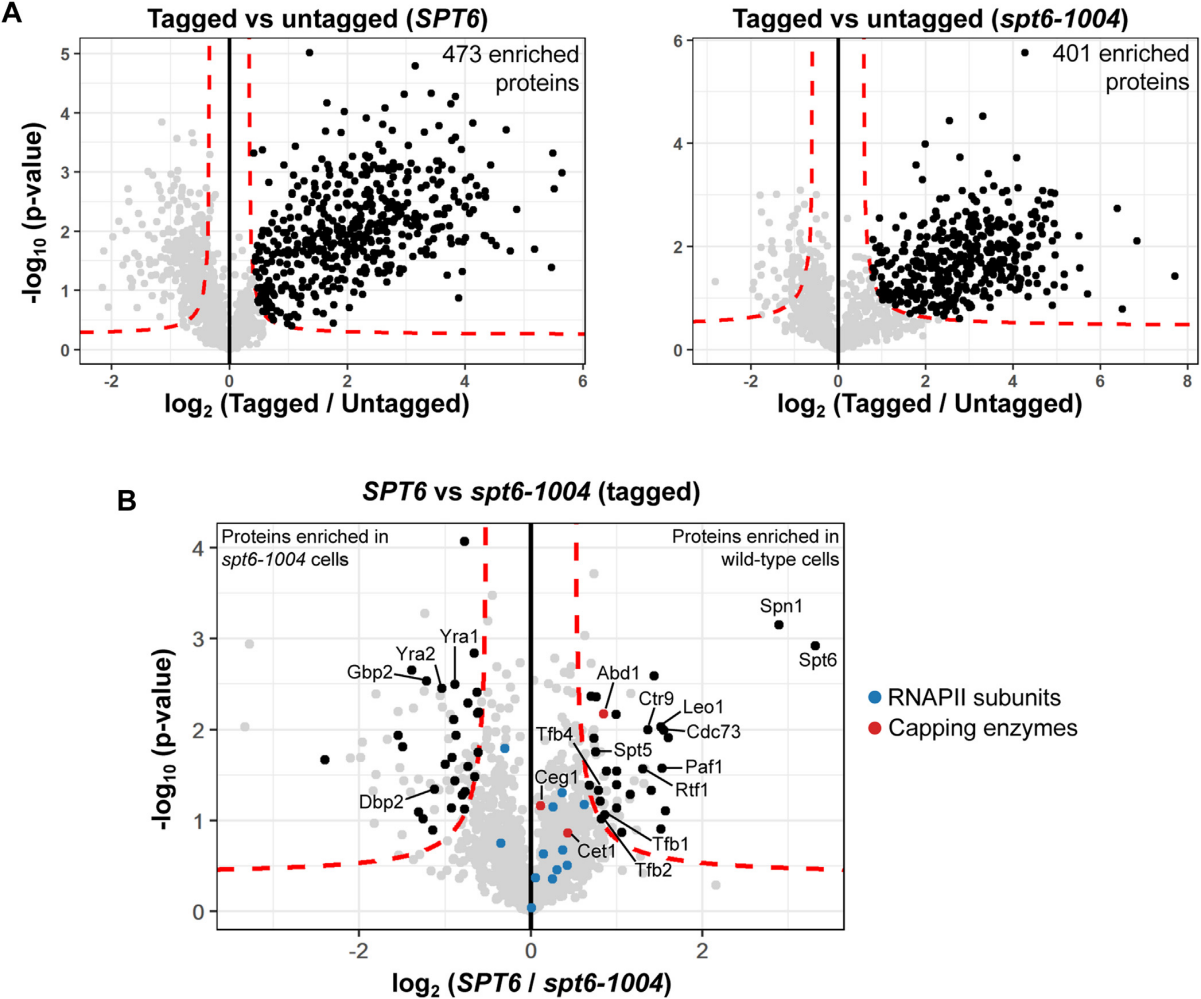
**Comparison of RNAPII-interacting proteins between wildtype and *spt6-1004* cells.***A*, volcano plot showing comparison of protein abundances between Rpb3-tagged and Rpb3-untagged samples in wildtype and *spt6-1004* cells. Each *dot* represents a single protein. The *dashed red line* indicates a permutation-based false discovery rate cutoff of 0.05. Proteins with a positive fold change and above this threshold are considered to be enriched in the tagged sample. *B*, volcano plot showing comparison of protein abundances between wildtype and *spt6-1004* cells in Rpb3-tagged strains. Each *dot* represents a single protein. The *dashed red line* indicates a permutation-based false discovery rate cutoff of 0.05. The 58 proteins that were identified to be differentially enriched, as well as enriched over the untagged control in either wildtype or *spt6-1004* cells, are highlighted in *black*. RNAPII subunits and capping enzymes are highlighted in *color*. RNAPII, RNA polymerase II.

To identify Rpb3-associated proteins that were specifically depleted or enriched in *spt6-1004* cells, the MS results were compared using Perseus software (44). First, protein abundances from Rpb3-tagged wildtype and Rpb3-tagged *spt6-1004* cells were each compared with the corresponding abundances from the untagged cells. Using a permutation-based false discovery rate (FDR) cutoff of 0.05, we identified 473 and 401 proteins (with more than one peptide detected for 352 and 308 proteins) that were enriched in Rpb3-tagged wildtype and Rpb3-tagged *spt6-1004* cells, respectively (Fig. 2*A*). Second, we compared protein abundances from Rpb3-tagged wildtype and Rpb3-tagged *spt6-1004* cells to identify proteins that were specifically enriched or depleted in *spt6-1004* and identified 137 such proteins (FDR <0.05). Of these, 58 proteins were also enriched over Rpb3-untagged samples based on our initial comparisons (Fig. 1*C* and Table 1). The remaining 79 proteins likely represent abundant nonspecific interactors, whose gene expression may be altered in *spt6-1004* based on previous transcriptional analysis (22).

**Table 1:** List of differential interactors identified by BioTAP-XL

Proteins enriched in WT		Proteins enriched in *spt6-1004*	
Protein	Fold change(WT/*spt6-1004*)	Protein	Fold change(WT/*spt6-1004*)
Transcription elongation factors		mRNA 3′ end processing and export	
Spt6	9.99	Gbp2	0.43
Spt5	1.69	Yra1	0.54
Spn1	7.45	Yra2	0.49
PAF complex subunits		Sub2	0.65
Ctr9	2.58	Hrp1	0.55
Rtf1	2.47	rRNA processing	
Paf1	2.89	Nog1	0.57
Cdc73	2.92	Nug1	0.54
Leo1	2.87	Erb1	0.63
General transcription factors		Nop6	0.66
Tfb1	1.82	Nop1	0.6
Tfb4	1.73	Rrp5	0.54
Tfb2	1.77	Dbp2	0.46
Toa1	1.7	Nsr1	0.59
Toa2	1.61	Tsr1	0.38
Mediator complex subunits		Ebp2	0.59
Srb7	2	Rpl8B	0.5
Med8	2.09	Others	
Cse2	2	Bur2	0.65
Med11	1.75	Chd1	0.6
Srb6	2.01	Tra1	0.53
Others		Nqm1	0.19
Abd1	1.8	Rep1	0.34
Wtm1	2.97	Rep2	0.36
Emg1	1.63	Orc4	0.45
Set2	1.84	Sis1	0.65
His5	1.67	Puf6	0.64
Tal1	2.65	Rex3	0.59
Ubp14	1.99	Tfc7	0.53
Iwr1	3.04	Gga1	0.4
Utp25	2.24	Trm2	0.42
Hpm1	2.87		
Erg27	2.71		

Our results from the MS analysis revealed some expected and some previously unknown differences in the RNAPII interactome between wildtype and *spt6-1004* cells. As expected, Spt6 was greatly decreased (a wildtype/*spt6-1004* decrease of approximately 10-fold) in *spt6-1004* (Fig. 2*B*) because of the depletion of the mutant Spt6 protein after the shift to 37 °C (Fig. 1*B*). The protein most decreased after Spt6 in *spt6-1004* was the TF Spn1 (a decrease of approximately 7.5-fold in *spt6-1004* as compared with wildtype), which is known to directly interact with Spt6 (32, 33). Recent evidence has shown that Spt6 is required for the association of Spn1 with RNAPII (36). We also observed decreased association of all PAF complex subunits with RNAPII in *spt6-1004* (Fig. 2*B*) (decreases ranging from 2.5- to 2.9-fold in *spt6-1004* as compared with wildtype), consistent with previous studies in yeast and *Drosophila*, which showed that Spt6 helps to recruit the PAF complex to actively transcribed regions (17, 31). The association of Spt5 with RNAPII was also decreased in *spt6-1004* (decreases of approximately 1.7-fold in *spt6-1004* as compared with wildtype) (Fig. 2*B*). A recent structure of the human transcription elongation complex shows Spt6 directly interacting with Spt5 while binding to RNAPII (14). Interestingly, we also identified the mRNA cap methyltransferase Abd1 as a protein decreased in RNAPII complexes in *spt6-1004* (fold decrease of approximately 1.8-fold in *spt6-1004* as compared with wildtype), unlike the two other capping enzymes, Cet1 and Ceg1 (Fig. 2*B*). There were also several proteins apparently enriched in *spt6-1004* cells, including mRNA export and rRNA processing factors (Table 1). In summary, our results suggest that Spt6 regulates the association of a number of transcription factors with RNAPII.

**Figure 3:**
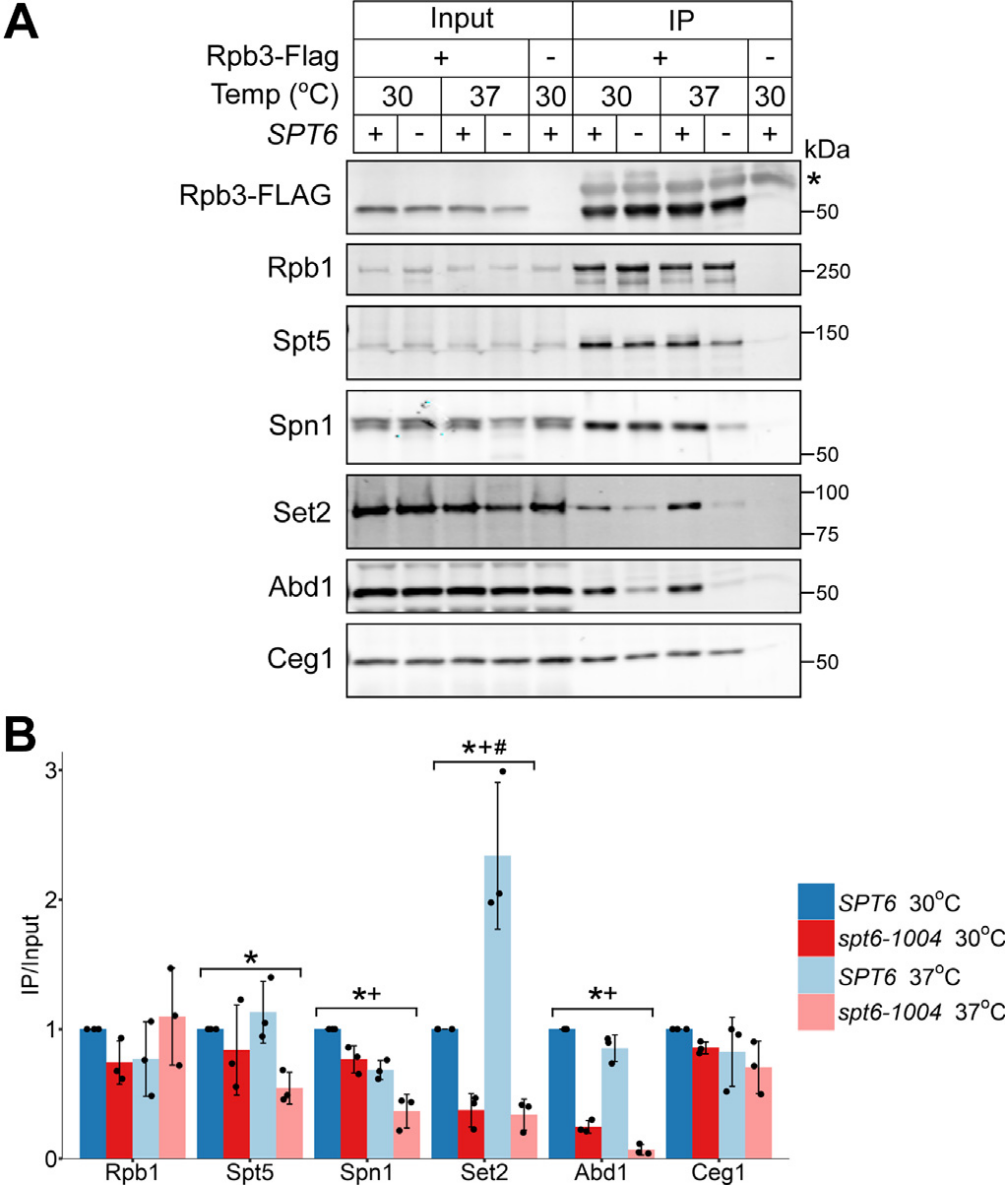
**Interactions of transcription elongation factors with Rpb3 in wildtype and *spt6-1004* cells.***A*, coimmunoprecipitation of the indicated proteins with FLAG-tagged Rpb3 in wildtype (+) and *spt6-1004* (−) cells before and after a shift to the nonpermissive temperature (37 °C). The *asterisk* (∗) indicates the detection of the IgG heavy chain. The images shown here are representative of three independent biological replicates. *B*, quantification of the Western blots shown in (*A*). The immunoprecipitated protein levels were normalized to both input levels of the same protein and immunoprecipitated Rpb3-FLAG levels. The error bars represent the mean ± standard deviation for three replicates. A two-way ANOVA test was used to determine if temperature and/or genotype significantly affected interaction with Rpb3. + represents factors where temperature was found to significantly affect Rpb3 interaction (*p* < 0.05). ∗ represents factors where genotype was found to significantly affect Rpb3 interaction (*p* < 0.05). # represents factors where a significant interaction between the effects of temperature and genotype was observed (*p* < 0.05).

To independently test for differential association of some of these proteins with RNAPII in wildtype and *spt6-1004* cells, we purified Rpb3 from uncrosslinked cells and tested for co-IP of differential interactors. The coimmunoprecipitations were done from cells harvested both before (30 °C) and after the temperature shift (37 °C). This helped to determine if the altered association of any factor might be primarily because of a specific defect in the *spt6-1004* mutant (30 °C) or depletion of Spt6 protein (37 °C). We observed that the association of Spt5 with Rpb3 was unchanged in *spt6-1004* cells at 30 °C but decreased at 37 °C as compared with wildtype cells (Fig. 3, *A* and *B*). This result agrees with our MS data and with previously published data in *Drosophila* cells (17), which shows that the level of Spt5 association with RNAPII is dependent on Spt6 protein levels. A similar observation was also made for Spn1 (Fig. 3, *A* and *B*), although Spn1 protein levels were decreased in *spt6-1004* cells at 37 °C, indicating that the reduced association of Spn1 with Rpb3 in *spt6-1004* could be explained in part by reduced Spn1 protein levels. However, a previous study has shown that the association of Spn1 with Rpb3 is dependent on Spt6 (36). The association of the H3K36 methyltransferase Set2 with Rpb3 was also decreased in *spt6-1004* cells at both temperatures (Fig. 3, *A* and *B*). This agrees with our previously published ChIP-Seq data (39), where we observed a modest decrease in Set2 recruitment to chromatin in *spt6-1004* cells at 30 °C. These co-IP experiments, then, support our MS data and have revealed a role for Spt6 regulating the level of Spt5 associated with RNAPII.

A novel finding from our MS data was the finding that Abd1 is dependent upon Spt6 for association with RNAPII. Our co-IP results also show reduced association of Abd1 with Rpb3 in *spt6-1004* cells, even though global Abd1 protein levels remained unaffected across all conditions tested (Fig. 3). Since Spt5 is involved in recruiting Abd1 to chromatin in *S. cerevisiae* (45), the observed differences in Abd1 binding could be due to decreased binding of Spt5 to RNAPII in *spt6-1004*. However, we observed that Abd1 association with Rpb3 was decreased in *spt6-1004* even at 30 °C where the association of Spt5 with Rpb3 was unaffected, suggesting that Spt6 regulates the Abd1–RNAPII interaction independently of Spt5. The co-IP of a different capping enzyme, Ceg1, with Rpb3 was unaffected in *spt6-1004* cells (Fig. 3), indicating that the altered Rpb3–Abd1 interaction was specific to Abd1 and is not a general property of capping enzymes. Thus, our data reveal a previously unknown role for Spt6 in promoting the association of Abd1 with RNAPII.

We also tested for co-IP of some of the proteins that were enriched in *spt6-1004* in our MS data (Dbp2, Chd1, and Iwr1). Unexpectedly, the association of these proteins with Rpb3 was decreased in *spt6-1004*, in contrast to the results from MS. Upon further investigation, Western blots suggested that the levels of some of these proteins were decreased in *spt6-1004* cells following a temperature shift (data not shown). While we cannot yet reconcile our MS data with these results, it is possible that, despite loss of total protein levels, a higher proportion of the remaining protein binds to RNAPII transiently, which can be captured only in the presence of the crosslinking that was used in the Bio-TAP-XL method.

### Genetic interactions of capping enzyme mutants with *spt6-1004*

**Figure 4:**
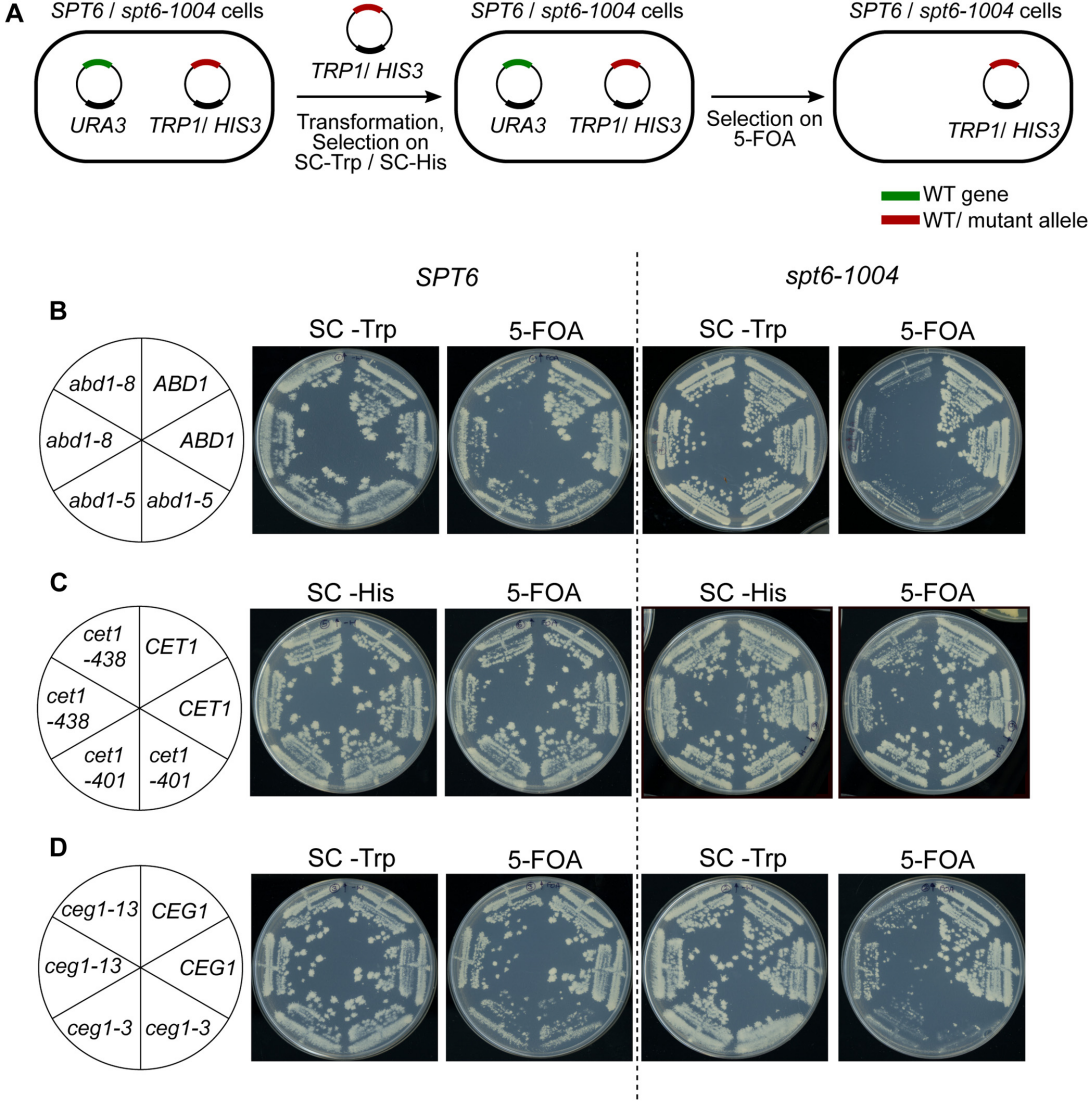
**Genetic interactions of capping enzyme mutants with *spt6-1004*.***A*, schematic outlining the plasmid shuffling strategy used to introduce capping enzyme mutants in wildtype and *spt6-1004* cells. *B*, growth of wildtype and *spt6-1004* cells expressing the indicated *ABD1* allele. The *abd1Δ* or *spt6-1004 abd1Δ* strains expressing wildtype *ABD1* on a *URA3* plasmid were transformed with the indicated *ABD1* allele on a *TRP1* plasmid. Individual transformants were replica plated on SC-Trp or 5-FOA medium and grown for 3 days. *C* and *D*, similar growth assays were conducted for *CET1* (*C*) and *CEG1* (*D*) alleles expressed on *HIS3* and *TRP1* plasmids, respectively.

Given our observation of decreased association of Abd1 with RNAPII in *spt6-1004*, we reasoned that *abd1* mutations might exacerbate some of the mutant phenotypes observed in *spt6-1004* strains. As *ABD1* is essential for viability, we tested this idea using two *abd1* temperature-sensitive mutations that cause defects in mRNA capping and transcription *in vivo* (46, 47). For both, we constructed *abd1 spt6-1004* double mutants by plasmid shuffling (Fig. 4*A*). Our results show that both *abd1-5* and *abd1-8* caused inviability at 30 °C when combined with *spt6-1004* (Fig. 4*B*), suggesting that fully functional *ABD1* is required when *SPT6* is mutated. To test if this is a general feature of capping enzyme mutants, we also combined *spt6-1004* with *cet1* and *ceg1* mutations using the same strategy outlined in Figure 4*A*. The mutations tested were *cet1-401*, *cet1-438*, *ceg1-3*, and *ceg1-13*, all of which are temperature sensitive and display defects in mRNA capping (48, 49). We observed that *cet1-438* and *cet1-401* are viable when combined with *spt6-1004* (Fig. 4*C*). On the other hand, *ceg1-3 spt6-1004* was inviable, and *ceg1-13 spt6-1004* grew poorly at 30 °C (Fig. 4*D*). Our results suggest that there is a functional interaction between Spt6 and mRNA capping, although not specific for Abd1. The greatly increased number of new transcription initiation sites in *spt6-1004* (22) might make the cells hypersensitive to impaired capping activity.

### Genome-wide localization of Abd1 and Spt5 in *spt6-1004*

Previous studies have indicated the requirement of RNAPII and Spt5 for the recruitment of Abd1 during transcription elongation (45, 50, 51). Our results suggest that Spt6 contributes to the recruitment of Abd1 to chromatin, either directly or possibly *via* decreased recruitment of RNAPII or Spt5. To test these ideas, we performed ChIP-Seq for Abd1-HA, Rpb1, and Spt5-V5 in wildtype and *spt6-1004* cells. For this experiment, cultures were grown at 30 °C, the temperature at which we observed defective Rpb3–Abd1 co-IP in *spt6-1004* despite unaltered Spt6 protein levels. To permit quantitative comparison of factor recruitment between different samples, we used chromatin from *Schizosaccharomyces pombe* for spike-in normalization (Fig. S2*A*). Each experiment was done in triplicate (biological replicates), and the replicates correlated well with one another (Fig. S2*B*).

In wildtype cells, ChIP-Seq of Abd1, Spt5, and RNAPII showed the expected patterns of occupancy. Abd1 levels peaked at the 5′ ends of genes at ∼160 bp downstream of the transcription start site, and occupancy at lower levels is observed throughout the gene body (Fig. S3*A*), consistent with a previous study (45). Abd1 occupancy showed a sharp decrease following the cleavage and polyadenylation site, suggesting that its association with gene bodies is transcription dependent. In support of its dependence on transcription, we also observed that Abd1 levels over gene bodies correlated with gene expression levels (Fig. S3*B*). The pattern of Spt5 occupancy over gene bodies mirrored that of RNAPII (Fig. S3, *B*, *C*, *E*, and *F*) (16). This is consistent with its association with RNAPII early in transcription and requirement for productive elongation (14, 18, 52). As expected, the levels of Spt5 and RNAPII occupancy over genes also correlated with their expression level (16) (Fig. S3, *E* and *F*).

**Figure 5:**
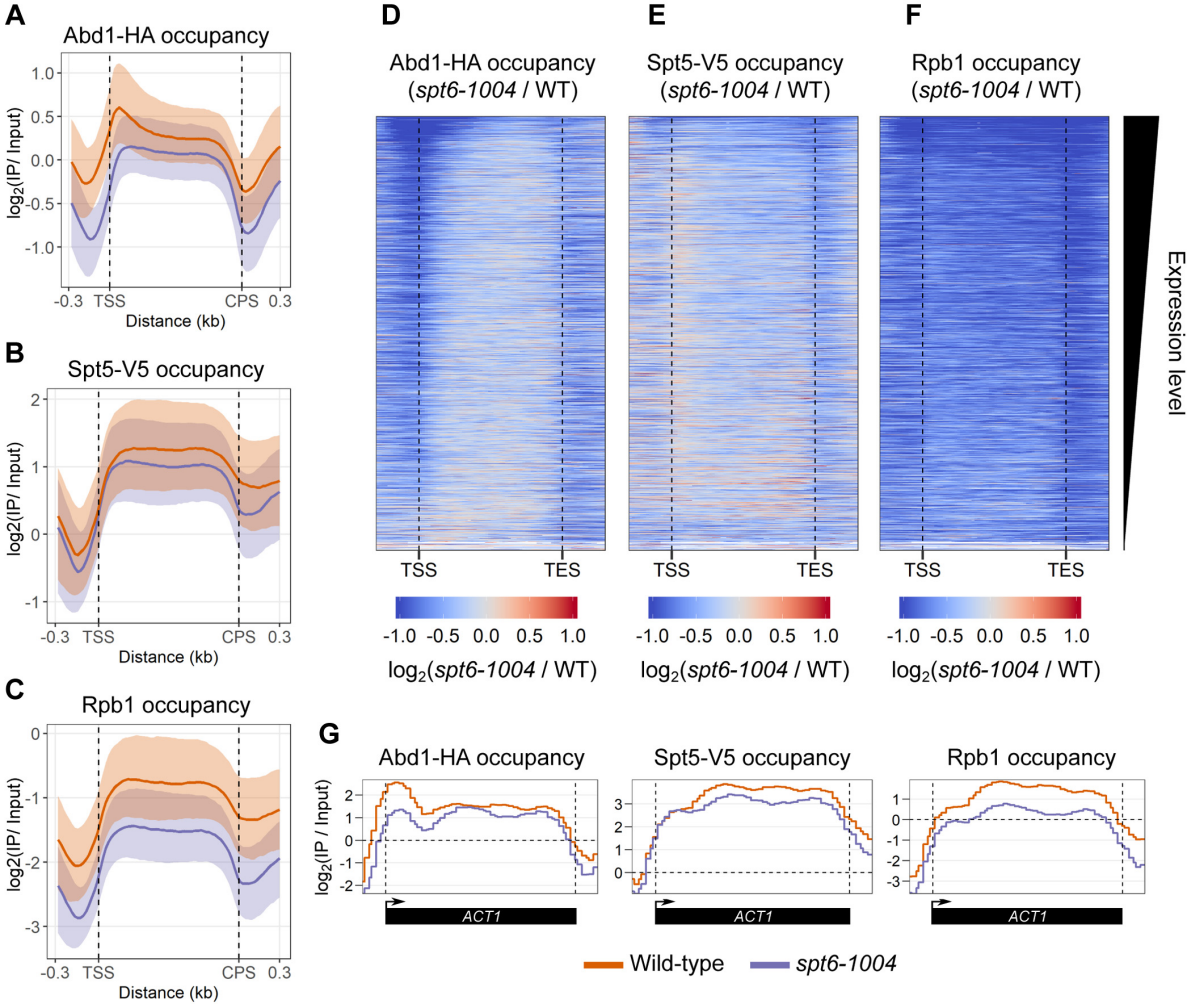
**Comparison of genome-wide occupancies of Abd1, Spt5, and RNAPII in wildtype and *spt6-1004*.***A*–*C*, metagene plots of fold change in Abd1-HA (*A*), Spt5-V5 (*B*), and Rpb1 (*C*) occupancy in wildtype and *spt6-1004* as compared with wildtype cells for 3522 nonoverlapping protein-coding genes. The occupancy in each genotype has been normalized to their respective inputs. The trace indicates the median occupancy at a given position. The shaded area represents the interquartile range. *D*–*F*, heat maps of fold change in Abd1-HA (*D*), Spt5-V5 (*E*), and Rpb1 (*F*) occupancy in *spt6-1004* as compared with wildtype cells for 3522 nonoverlapping protein-coding genes. The occupancy in each genotype has been normalized to their respective inputs. All values that had a log fold change of below −1 or above 1 are set to −1 and 1, respectively. The genes are arranged in decreasing order of expression level as determined from RNA-Seq of wildtype cells (36). *G*, representative traces of Abd1-HA, Spt5-V5, and Rpb1 occupancy in wildtype and *spt6-1004* cells at the highly transcribed *ACT1* gene. RNAPII, RNA polymerase II.

In an *spt6-1004* mutant, we saw striking differences from wildtype. First, we observed a global decrease in RNAPII occupancy in *spt6-1004*, consistent with previous results (39) (Fig. 5, *C* and *F*). Second, we also observed a global decrease in both Abd1 and Spt5 ChIP-Seq levels (Fig. 5, *A*, *B*, *D*, and *E*). These are likely caused, at least in part, by the decreased levels of RNAPII in *spt6-1004*, as recruitment of both factors to chromatin is dependent on RNAPII (14, 50, 52). However, although RNAPII levels were decreased uniformly over transcribed regions in *spt6-1004* (Fig. 5*C*), the levels of Abd1 and Spt5 did not follow the same pattern. Abd1 levels showed a greater decrease over the 5′ ends of genes as compared with the decrease over gene bodies, with the effect greatest over the most highly transcribed genes (Fig. 5, *A* and *D*). In contrast, the levels of Spt5 were more affected over the gene bodies as compared with the 5′ ends of genes (Fig. 5, *B* and *E*). This suggests that Spt6 is required for maintaining the levels of Abd1 over the 5′ ends of genes and of Spt5, after its recruitment to the transcription elongation complex. Finally, the loss of Abd1, Spt5, and RNAPII occupancy in *spt6-1004* was more prominent at highly expressed genes (Figs. 5, *D*–*F* and S4), highlighting the importance of Spt6 during transcription, and suggesting a greater dependence on Spt6 for Abd1 recruitment at highly transcribed genes. Examples of occupancy levels of Abd1, Spt5, and RNAPII at the highly transcribed *ACT1* gene are shown in Figure 5*G*. In summary, our results suggest that Spt6 regulates Abd1 and Spt5 recruitment over transcribed regions.

## Discussion

We have identified a role for Spt6 in maintaining the association of TFs with RNAPII. The RNAPII-interacting proteins that we have identified in wildtype cells are similar to those identified in previous studies (53, 54), indicating that BioTAP-XL can be used to successfully purify protein complexes in yeast. Of the RNAPII-interacting proteins detected, we identified 58 that were specifically depleted or enriched in RNAPII complexes in an *spt6-1004* mutant. These included factors previously known to interact with Spt6 (such as Spt5 and Spn1) as well as factors whose relationship with Spt6 was previously unknown, most notably Abd1. Our results suggest that the altered accumulation of some of these factors is due to their reduced protein levels in *spt6-1004*, which may be due to changes in either gene expression or protein stability. We followed up on one of the proteins whose steady-state level remained unchanged in *spt6-1004*—the mRNA cap methyltransferase Abd1—and showed that the occupancy of Abd1 on chromatin in *spt6-1004* is decreased genomewide. We also identified a decreased occupancy for Spt5 genomewide, revealing another requirement for Spt6 in regulating transcriptional processes.

We showed that the levels of Abd1 are most decreased at the 5′ ends of genes in the *spt6-1004* mutant. Two plausible models can explain this observation. In the first model, Spt6 plays a role in recruiting Abd1 specifically at the 5′ end. While Abd1 occupancy depends on Spt5 throughout the gene, it may in addition depend on Spt6 levels toward the 5′ ends of genes. This would explain why we see a greater defect in Abd1 recruitment at the 5′ end even when the Spt5 recruitment defect is lowest in this region. While Spt6 and Abd1 have been shown to be part of the same protein complex by co-IP studies (55), it remains to be determined if there is a direct interaction between the two proteins. A second possibility is that levels of Ser-5 phosphorylated C-terminal domain of Rpb1, which is required for normal recruitment of Abd1 (50), might be decreased in *spt6-1004*. In support of this hypothesis, in the *spt6-1004* mutant, we also observed decreased RNAPII association of Tfb1, Tfb2, and Tfb4 (Fig. 2*B*), which are components of the TFIIH complex that contains the Kin28 kinase that phosphorylates the Ser-5 residue of the C-terminal domain. Hence, although required for Abd1 recruitment, the role of Spt6 in this process might be through an indirect effect on transcription (46).

By ChIP-Seq, we also observed that RNAPII and Spt5 occupancies are decreased over transcribed regions genomewide in *spt6-1004*. Our previous data also showed a global decrease in RNAPII ChIP-Seq signal in *spt6-1004* (39), although to a lesser extent. The higher magnitude observed here may be due to the presence of the V5 tag on Spt5 in the same strain, leading to genetic interactions between *SPT5* and *spt6-1004* that impair transcription. However, we do not observe any effect of *SPT5-V5* on the temperature sensitivity or Spt^−^ phenotypes of *spt6-1004*. While RNAPII levels show a uniform decrease over gene bodies, the levels of Spt5 decrease more at the 3′ ends of genes as compared with the 5′ ends, indicating that Spt6 might be required for continued association of Spt5 with RNAPII during transcription elongation. This agrees with previously published data showing reduced recruitment of Spt5 to the *Hsp70* gene body upon knockdown of *SPT6* in *Drosophila* cells (17). This is also in line with the structure of the human transcription elongation complex, where part of Spt5 is sandwiched between RNAPII and Spt6 (14). Loss of Spt6 might allow dissociation of Spt5, leading to the lower levels of RNAPII observed genomewide in *spt6-1004*.

The decreased association of Abd1 over transcribed regions in *spt6-1004* likely has functional consequences. This is supported by our genetic data showing loss of viability upon combining *abd1* mutations with *spt6-1004*. First, decreased association of Abd1 can affect mRNA capping, possibly leading to the production of transcripts that have nonmethylated mRNA caps. Such incompletely capped mRNAs are observed even in wildtype cells under stress conditions, and the exonucleases Rai1 and Dxo1 are involved in the recognition and degradation of these transcripts (56, 57, 58). This indicates that inefficient mRNA capping can occur under physiological conditions and that the cell has developed quality control mechanisms to prevent the accumulation of aberrantly capped transcripts. Second, the decreased association of Abd1 over gene bodies can affect transcription. Highly transcribed genes show the highest Abd1 recruitment defect in *spt6-1004* cells (Figs. 5*D* and S4*A*). A previous study has shown a subset of highly transcribed genes in yeast to be dependent on Abd1 but not Ceg1 for promoter clearance of RNAPII and stabilization of RNAPII following its recruitment to chromatin (46). Similarly, Abd1 has also been observed to positively promote transcription in human cells (59). Transcription at highly transcribed genes may be affected the most because of reduced Abd1 recruitment in *spt6-1004*. Future experiments will help determine if the reduced interaction of Abd1 with RNAPII may be contributing to some of the phenotypes observed in *spt6-1004*.

## Experimental procedures

### Strains and media

All *S. cerevisiae* and *S. pombe* strains used are listed in Table S1. All plasmids used are listed in Table S2. All oligos used for strain constructions are listed in Table S3. All *S. cerevisiae* strains were grown in yeast extract–peptone–dextrose (YPD) medium (1% yeast extract, 2% peptone, and 2% glucose) unless mentioned otherwise. All *S. pombe* strains were grown in yeast extract with supplements medium (0.5% yeast extract, 3% glucose, and 225 mg/l each of adenine, histidine, leucine, uracil, and lysine). For experiments involving temperature shifts, cells were first grown to an absorbance of ≈0.6 (∼2 × 10^7^ cells/ml) at 600 nm at 30 °C. One volume of culture was mixed with an equal volume of YPD at 42 °C. The cells were then grown at 37 °C for 80 min. All strains were constructed by standard yeast crosses or transformations (60). For tagging Rpb3 with the BioTAP tag, the DNA sequence encoding the tag was amplified from plasmid FB2729, and the PCR fragment was used to transform yeast strain FY57, resulting in integration into the *S. cerevisiae* genome, replacing the stop codon in the *RPB3* gene. Tagged *RPB3* was introduced in *spt6-1004* through a genetic cross with FY3332. For tagging *ABD1* with 3xHA, the DNA encoding the tag was amplified from the plasmid pFA6a-3xHA-kanMX6 (61), and the PCR fragment was transformed into a wildtype yeast strain, resulting in integration into the *S. cerevisiae* genome, replacing the stop codon in the *ABD1* gene. *SPT5-3xV5* and *spt6-1004* were introduced in the tagged *ABD1* strain through standard genetic crosses. Wildtype or mutated versions of genes encoding capping enzymes were introduced into yeast strains by plasmid shuffling, using plasmids CE113, CE333, and CE339 for *CET1*; RGBO5, RGBO6, and RGBO7 for *CEG1*; and RGBO8, RGBO9, and RGBO10 for *ABD1*.

### Purification of RNAPII complexes by BioTAP-XL and analysis by MS

BioTAP-XL was done as previously described (41), with modifications to adapt the protocol from *D. melanogaster* cells to *S. cerevisiae* cells. Yeast strains FY3330, FY3331, FY57, and FY3332 were grown in 1.5 l of YPD supplemented with 6 μM biotin at 30 °C to an absorbance of ≈0.6 at 600 nm. This was followed by addition of 1.5 l of YPD (supplemented with 6 μM biotin) at 42 °C and growth at 37 °C for 80 min. The yeast cultures were then rapidly cooled by transferring the cells to a flask with 0.3× volume of media at 4 °C, and FA was immediately added to a final concentration of 1%. The cultures were incubated with shaking at room temperature for 30 min. Glycine was then added to a final concentration of 125 mM, and the incubation was continued for 5 min. The cells were collected by filtration onto a 0.45-μm nitrocellulose membrane filter. The cells were then scraped off the filter, inserted into a syringe, and expelled into liquid nitrogen. The frozen cells were lysed in a mixer mill for six cycles, 3 min each at 15 Hz, with incubation in liquid nitrogen between each cycle, to form a “grindate.” The grindate was suspended in 12 ml of cold FA lysis buffer (50 mM Hepes–KOH, pH 7.5, 140 mM NaCl, 1 mM EDTA, 0.1% sodium deoxycholate, 0.1% Triton X-100, 0.05% SDS, 2 μg/ml leupeptin, 2 μg/ml pepstatin, and 1 mM PMSF) and divided equally between 15 tubes. The sample in each tube was pelleted by centrifugation, resuspended in 640 μl of FA lysis buffer, and sonicated in a Qsonica machine for 25 min (30 s on, 30 s off, 70% amplitude). The sonicated samples were centrifuged at 12,500 rpm for 30 min at 4 °C. The supernatants from individual tubes were pooled and incubated with 1.8 ml of IgG beads (prewashed with FA lysis buffer) overnight at 4 °C.

Following the IP, the beads were washed three times with radioimmunoprecipitation buffer (10 mM Tris–HCl, pH 8.0, 140 mM NaCl, 1 mM EDTA, 1% Triton X-100, and 0.1% SDS). For each wash, the resuspended beads were incubated at 4 °C for 10 min with end-over-end rotation. The beads were then washed once with TEN140 buffer (10 mM Tris–HCl, pH 8.0, 140 mM NaCl, and 1 mM EDTA) with end-over-end rotation for 2 min at room temperature. The protein complexes were eluted twice by incubation with 20 ml of freshly made IgG elution buffer (100 mM Tris–HCl, pH 8.0, 200 mM NaCl, 6 M urea, and 0.2% SDS) with end-over-end rotation for 1 h at room temperature. The eluted sample was concentrated in an Amicon Ultra-15 column (10-kDa cutoff; Millipore), and buffer exchange was done four times with 12 ml of TEN140 buffer. The resulting 1 ml of concentrated sample was brought up to 2.8 ml with radioimmunoprecipitation buffer and incubated with 1 ml of streptavidin agarose beads (prewashed with TEN140 buffer).

Following the affinity pulldown, the beads were washed once with TEN140 buffer with 0.1% Triton-X 100, twice with IgG elution buffer, twice with IgG elution buffer without SDS, and once with TEN140 buffer for 5 min each at 4 °C. The beads were then washed seven times with 50 mM ammonium bicarbonate for 5 min each at room temperature and then suspended in 800 μl of 50 mM ammonium bicarbonate and split evenly between two tubes. About 10 μl of trypsin was added to one tube, which was then incubated overnight at 37 °C with end-over-end rotation. The beads in the other tube were boiled for 25 min in reverse crosslinking buffer (250 mM Tris–HCl, pH 8.8, 2% SDS, and 0.5 M β-mercaptoethanol), and the resulting supernatant was stored at −70 °C.

One microliter of 100% formic acid was added to the trypsinization reaction. The beads were centrifuged at 3000 rpm for 5 min at 25 °C, and the supernatant containing the digested peptides was transferred to a new tube. The beads were washed three times with a solution of 25% acetonitrile and 0.1% formic acid, and the supernatants after each wash were pooled with the initial supernatant. A C18 spin tip was equilibrated with 50 μl of 100% acetonitrile and twice with 50 μl of 0.1% trifluoroacetic acid. The peptides were passed twice through the C18 spin tip, which was then washed twice with 0.1% trifluoroacetic acid. The peptides were then eluted from the resin once with 30 μl of 50% acetonitrile and once with 30 μl of 100% acetonitrile. The eluted peptides were dried completely in a SpeedVac vacuum concentrator, and the dried peptides were stored at −70 °C until analysis by MS.

MS analysis was conducted by Ryan Kunz at the Taplin MS facility at Harvard Medical School. In all, eight samples were submitted—four genotypes (*SPT6*, *RPB3*-untagged; *SPT6*, *RPB3*-tagged; *spt6-1004*, *RPB3*-untagged; and *spt6-1004*; *RPB3*-tagged) in biological duplicates. The enzyme used to cleave the proteins was trypsin that cleaves peptides on the C-terminal side of lysines and arginines. The peptides from each sample were labeled with TMTs (TMT-127N, TMT-131 for *SPT6*, *RPB3*-untagged samples; TMT-126 and TMT-130 for *SPT6*, *RPB3*-tagged samples; TMT-129N, TMT-129C for *spt6-1004*, *RPB3*-untagged samples; and TMT-127C, TMT-128 for *spt6-1004*, *RPB3*-tagged samples). Quantification of peptide abundances among the different samples was done at the MS3 stage (43). The pooled peptides were run on an Orbitrap Fusion mass spectrometer. Peptides were separated using a gradient of 3 to 23% acetonitrile in 0.125% formic acid over 180 min. The peptides were detected (MS1) and quantified (MS3) in the Orbitrap and sequenced (MS2) in the ion trap. The maximum number of miscleavage sites permitted was 2. The following static modifications were considered—carboxyamidomethylation of cysteine (+57.0214637236 Da) and TMT labeling of N-terminal residues and lysines (+229.162932 Da). The variable modification considered was the oxidation of methionine (+15.9949146221 Da). The mass tolerance for precursor ions was 50 ppm, and the mass tolerance for fragment ions was 1 Da.

### Analysis of MS data

MS2 spectra were searched using the SEQUEST algorithm against a composite database derived from the yeast proteome. The composite database was composed of the *S. cerevisiae* reference proteome (downloaded from UniProt on December 3, 2014), common contaminants, and all sequences in reverse. Total number of sequence entries was 13,720. Peptide spectral matches were filtered to a 1% FDR using the target-decoy strategy combined with linear discriminant analysis. The proteins were filtered to a <1% FDR. Proteins were quantified from peptides with a summed SN threshold of ≥200 and MS2 isolation specificity of 0.5.

Following reporter ion quantification, the peptide intensities for each protein were summed and normalized to the total peptide intensity in each sample. The summed and normalized peptide intensities are presented in Table S4. The data were processed using Perseus software (44). The normalized protein abundances for each protein were log transformed, and missing values (for proteins that had summed peptide intensity = 0) were imputed from a normal distribution. The resulting log-transformed protein abundances were used for the calculation of Pearson correlations between samples in Figure S1.

For the calculation of fold changes between genotypes, the samples were grouped by genotype, and a *t* test was conducted to compare the signal for each protein between two samples of interest. The *t* test gives the difference of the means of biological replicates between the two genotypes being compared. A permutation-based FDR threshold was calculated and used to identify proteins as being significantly enriched in one sample *versus* the other. Volcano plots were generated using custom R scripts.

### Co-IP assays

For each co-IP experiment, 50 ml of yeast cells at an absorbance of ≈0.6 (∼2 × 10^7^ cells/ml) at 600 nm were harvested. The cell pellets were suspended in 500 μl of IP buffer (20 mM Hepes–KOH, pH 7.6, 125 mM potassium acetate, 1 mM EDTA, 20% glycerol, 1 mM DTT, 1% NP-40, and 1× Sigma protease inhibitor cocktail). One milliliter of acid-washed glass beads was added to each tube, and the cells were lysed by bead beating for 8 min with incubation on ice for 3 min after each minute. The resulting lysate was centrifuged at 12,500 rpm for 10 min at 4 °C. The supernatant was transferred to a new tube. Protein concentrations in the whole cell lysates were measured by Bradford assay (62). One milligram of protein in a final volume of 500 μl was incubated with 20 μl of anti-Flag agarose beads (Sigma) prewashed with IP buffer. The IP was carried out for 2 h at 4 °C. The beads were then washed thrice with 500 μl of IP buffer following which the beads were boiled for 5 min in 50 μl of modified SDS buffer (60 mM Tris–HCl, pH 6.8, 4% β-mercaptoethanol, 4% SDS, 0.01% bromophenol blue, and 20% glycerol). The beads were centrifuged at 12,500 rpm for 1 min at room temperature, and 15 μl of the supernatant was loaded on SDS-PAGE gels for Western blotting.

### Western blotting and antibodies

Whole cell extracts from yeast were prepared as described previously (39). Primary antibodies used for Western blotting were anti-Set2 (1:8000; provided by Brian Strahl), anti-Abd1 (1:1000; provided by Stephen Buratowski), anti-Ceg1 (1:2000; provided by Stephen Buratowski), anti-Spn1 (1:8000; provided by Laurie Stargell), anti-Spt6 (1:10,000; provided by Tim Formosa), anti-Rpb1 (1:1000; Millipore; 8WG16), anti-HA (1:5000; Abcam; ab9110), anti-Flag (1:5000; Sigma; F3165), anti-V5 (1:5000; Invitrogen; R960-25), anti-Pgk1 (1:10,000; Life Technologies; 459250), and anti-Act1 (1:10,000; Abcam; ab8224). Secondary antibodies used were goat anti-rabbit IgG (1:10,000; Licor IRDye 680RD) and goat antimouse IgG (1:20,000; Licor; 800CW). Quantification of Western blots was done using ImageStudio (LI-COR Biosciences) software.

### ChIP

ChIP-Seq library preparation was done as described previously (39), with minor modifications. All samples were grown in triplicate, each starting from an independent yeast colony (biological replicates). Each sample was spiked in with *S. pombe* chromatin from strains FWP566 and FWP485 to a final concentration of 7.5% for each strain prior to the IP step. About 5 μl of anti-HA antibody (Abcam; ab9110) per 500 μg of chromatin, 7.5 μl of anti-V5 antibody (Invitrogen; R960-25) per 500 μg of chromatin, and 10 μl of anti-Rpb1 antibody (Millipore; 8WG16) per 500 μg of chromatin were used for IP of Abd1-HA, Spt5-V5, and Rpb1, respectively. The libraries were sequenced on an Illumina NextSeq platform.

### ChIP-Seq data analysis

Demultiplexing, alignment, spike-in normalization, and generation of coverage files from FASTQ files were done as described previously (39). Briefly, reads were demultiplexed using the fastq-multx command (https://github.com/brwnj/fastq-multx), and low-quality bases were trimmed using the cutadapt command (63) (threshold quality score of 20). The trimmed reads were aligned to a reference genome consisting of the *S. cerevisiae* + *S. pombe* genomes using Bowtie2 (64), and reads with a mapping quality score of greater than 3 were retained for further analysis. Crosscorrelation was used to determine ChIP-Seq fragment lengths (65). Since the replicates correlated well with one another, the BAM files for the replicates were merged prior to generating coverage files for plotting. Coverage files were produced using (66) igvtools while extending reads by the average fragment length in each sample. The coverage was spike-in normalized as previously described (67), while correcting for variations in the input samples. Ratios of spike-in normalized IP and input coverage files were calculated, and matrices suitable for plotting heat maps and metagenes were generated using commands from the deeptools suite (68). Metagenes and heat maps were generated using custom R scripts.

## Data availability

The RNA-Seq and ChIP-Seq datasets are available in the Gene Expression Omnibus repository, accession number GSE171953. They can be accessed at https://www.ncbi.nlm.nih.gov/geo/query/acc.cgi?acc=GSE171953. The MS proteomics data have been deposited to the ProteomeXchange Consortium *via* the PRIDE [1] partner repository with the dataset identifier PXD027708 and 10.6019/PXD027708. All other relevant data supporting the key findings of this study are available within this article and its supporting information.

## Supporting information

This article contains supporting information (36, 61).

## Conflict of interest

The authors declare that they have no conflicts of interest with the contents of this article.
