# Supplementary material for: The histone chaperone Spt6 is required for normal recruitment of the capping enzyme Abd1 to transcribed regions
Source: J Biol Chem. 2021 Sep 17;297(4):101205. doi: 10.1016/j.jbc.2021.101205 (PMC8511950; doi:10.1016/j.jbc.2021.101205)
Supplement: Tables S1–S3 and Figures S1–S4 [file mmc1.pdf]

Supplementary information

The histone chaperone Spt6 is required for normal recruitment of the capping enzyme Abd1 to transcribed regions

Rajaraman Gopalakrishnan and Fred Winston

Table S1 – Yeast strains
Table S2 – Plasmids
Table S3 – Oligonucleotides
Table S4 – List of proteins identified by BioTAP-Xl

Figure S1 – Reproducibility of mass spectrometry data
Figure S2 – Quality control analysis of ChIP-seq data
Figure S3 – Genome-wide recruitment of Abd1, Spt5, and RNAPII to transcribed regions in wild-type cells
Figure S4 – Occupancy of Abd1, Spt5, and RNAPII over transcribed regions binned by their expression level in wild-type cells

Table S1. List of yeast strains

Strain	Genotype	Species	Source
RGC11	*MAT***a** *his4-912δ lys2-128δ ura3-52 RPB3-BIOTAP::kanMX*	*S. cerevisiae*	This study
RGC14	*MAT***a** *his4-912δ lys2-128δ ura3-52 RPB3-BIOTAP::kanMX spt6-1004*	*S. cerevisiae*	This study
FY57	*MAT***a** *his4-912δ lys2-128δ ura3-52*	*S. cerevisiae*	Winston Lab
RGC42	*MAT***a** *his4-912δ lys2-128δ ura3-52 spt6-1004*	*S. cerevisiae*	Winston Lab (Natalia Reim)
FY2912	*MAT*α *ura3-52 his4-912δ lys2-128δ Rpb3-3xFlag-NatMX*	*S. cerevisiae*	Winston Lab
FY2913	*MAT*α *ura3-52 his4-912δ lys2-128δ Rpb3-3xFlag-NatMX spt6-1004*	*S. cerevisiae*	Winston Lab
FY56	*MAT*α *ura3-52 his4-912δ lys2-128δ*	*S. cerevisiae*	Winston Lab
RGC549	*MAT***a** *his3Δ200 lys2-128δ ura3-52 leu2Δ1 ABD1-HA-KanMX SPT5-3xV5*	*S. cerevisiae*	This study
RGC550	*MAT***a** *his3Δ200 lys2-128δ ura3-52 leu2Δ1 ABD1-HA-KanMX SPT5-3xV5 spt6-1004*	*S. cerevisiae*	This study
RGC537	*MAT***a** *his3Δ200 lys2-128δ ura3-52 leu2Δ1 trp1Δ63 cet1Δ1::TRP1 [pRS316-CET1]*	*S. cerevisiae*	This study
RGC506	*MAT***a** *his3Δ200 lys2-128δ ura3-52 leu2Δ1 trp1Δ63 spt6-1004 cet1Δ1::TRP1 [pRS316-CET1]*	*S. cerevisiae*	This study
RGC511	*MAT***a** *his3Δ200 lys2-128δ ura3-52 leu2Δ1 trp1Δ63 ceg1Δ::kanMX [pRS316-CEG1]*	*S. cerevisiae*	This study
RGC507	*MAT***a** *his3Δ200 lys2-128δ ura3-52 leu2Δ1 trp1Δ63 spt6-1004 ceg1Δ::kanMX [pRS316-CEG1]*	*S. cerevisiae*	This study
RGC512	*MAT*α *lys2-128δ ura3-52 leu2Δ1 trp1Δ63 abd1Δ::kanMX [pRS316-ABD1]*	*S. cerevisiae*	This study
RGC510	*MAT*α *lys2-128δ ura3-52 leu2Δ1 trp1Δ63 spt6-1004 abd1Δ::kanMX [pRS316-ABD1]*	*S. cerevisiae*	This study

Table S1. List of yeast strains

**Strain**	**Genotype**	**Species**	**Source**
FWP566	*h- set2-3xHA-NatMX*	*S. pombe*	Winston lab
FWP485	*h- spt5::spt5+-3xV5-IAA17-kanMX6 ade6::ade6+-Padh15-skp1-OsTIR1-natMX6-Padh15-skp1-AtTIR1-2NLS-9myc ura4-D18*	*S. pombe*	Winston lab

Table S2. List of plasmids

**Strain**	**Description**	**Host**	**Source**
FB2729	*pFA6a + 6xGLY - Biotin - 2x TEV - 2x protein A*	DH5α	Winston Lab
RGBO5	*TRP1 CEN ampR CEG1*	DH5α	Beate Schwer
RGBO6	*TRP1 CEN ampR ceg1-3*	DH5α	Beate Schwer
RGBO7	*TRP1 CEN ampR ceg1-13*	DH5α	Beate Schwer
RGBO8	*TRP1 CEN ampR ABD1*	DH5α	Beate Schwer
RGBO9	*TRP1 CEN ampR abd1-5*	DH5α	Beate Schwer
RGBO10	*TRP1 CEN ampR abd1-8*	DH5α	Beate Schwer
CE113	*HIS3 CEN ampR CET1*	DH5α	Stephen Buratowski
CE333	*HIS3 CEN ampR cet1-401*	DH5α	Stephen Buratowski
CE339	*HIS3 CEN ampR cet1-438*	DH5α	Stephen Buratowski
FB2317	pFA6a-3xHA-kanMX6	DH5α	Longtine et al. (1998)

Table S3. List of oligonucleotides

Name	Gene	Sequence	Purpose
FO9884	*RPB3-FB2729*	AATGGGTAATACTGGATCAGGAGGG TATGATAATGCTTGGCGGATCCCCGG GTTAATTAA	FP for tagging *RPB3* with BioTAP-KanMX
FO9885	*RPB3-FB2729*	GTTCACTTGTTTTTTTTCCTCTATTAC GCCCACTTGAGAAGAATTCGAGCTCG TTTAAAC	RP for tagging *RPB3* with BioTAP-KanMX
RG108	*TAP-HIS5*	GGTAAAACAGTATATCGAACCGGAA AGCGTAAAGCCCAACGGTCGACGGA TCCCCGGGTT	FP to tag *ABD1* with TAP
RG109	*TAP-HIS5*	ATGCTTTATAGTAGGGTTATTGTTTCT ATTCATTTTTATTTCGATGAATTCGA GCTCGTT	RP to tag *ABD1* with TAP
RG91	*ABD1-pFA6a*	GGTAAAACAGTATATCGAACCGGAA AGCGTAAAGCCCAACCGGATCCCCG GGTTAATTAA	FP for tagging *ABD1* with 3x HA
RG92	*ABD1-pFA6a*	ATGCTTTATAGTAGGGTTATTGTTTCT ATTCATTTTTATTGAATTCGAGCTCGT TTAAAC	RP for tagging *ABD1* with 3x HA
FO9181	*ADH1*	TCCTTGTTTCTTTTTCTGCAC	FP for ADH1 5' ChIP
FO9182	*ADH1*	GAGATAGTTGATTGTATGCTTGG	RP for ADH1 5' ChIP
FO955	*ChrV*	GGCTGTCAGAATATGGGGCCGTAGT A	FP for amplifying untranscribed region on ChrV for ChIP
FO956	*ChrV*	CACCCCGAAGCTGCTTTCACAATAC	RP for amplifying untranscribed region on ChrV for ChIP
RG114	*PDC1*	ATGTCTGAAATTACTTTGGGTA	FP ChIP 5' region of PDC1
RG115	*PDC1*	AATTCGTTGGCGTTACCAGC	RP ChIP 5' region of PDC1
RG123	*ILV5*	ACCCTACCAGCAATATAAGT	FP for ChIP 5' region
RG124	*ILV5*	GGGTGGCCAAAGCAAAGGTT	RP for ChIP 5' region

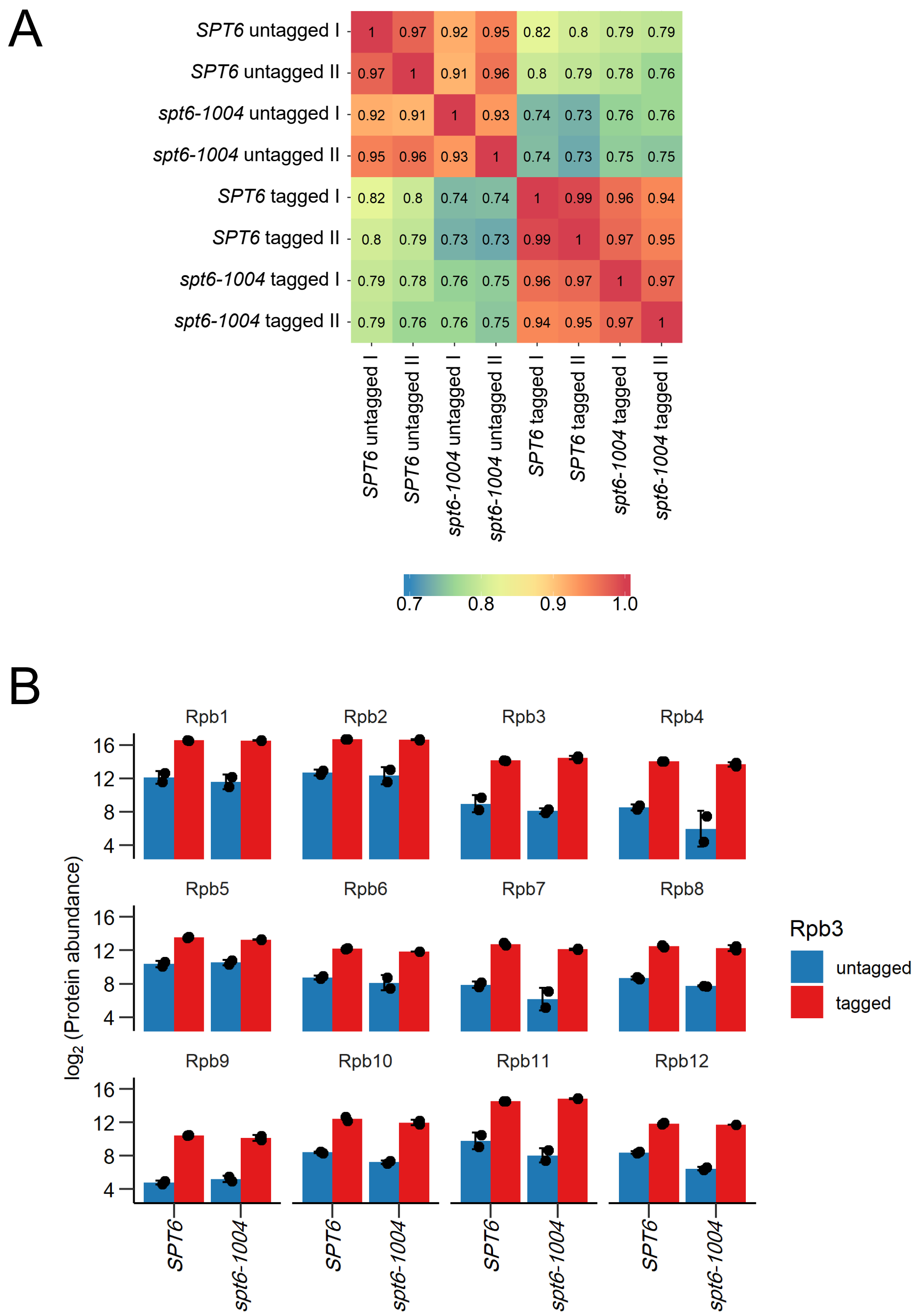

Supplementary Figure 1. Control analyses for mass spectrometry data.
(A) Heatmap showing pearson correlation coefficients of average peptide intensities for each protein across all samples analyzed. The moderately high correlation (Pearson R > 0.7) betwen the tagged and untagged samples suggests the presence of a large number of non-specifically associated proteins. (B) Normalized protein abundances of RNAPII subunits in tagged and untagged wild-type and *spt6-1004* cells.

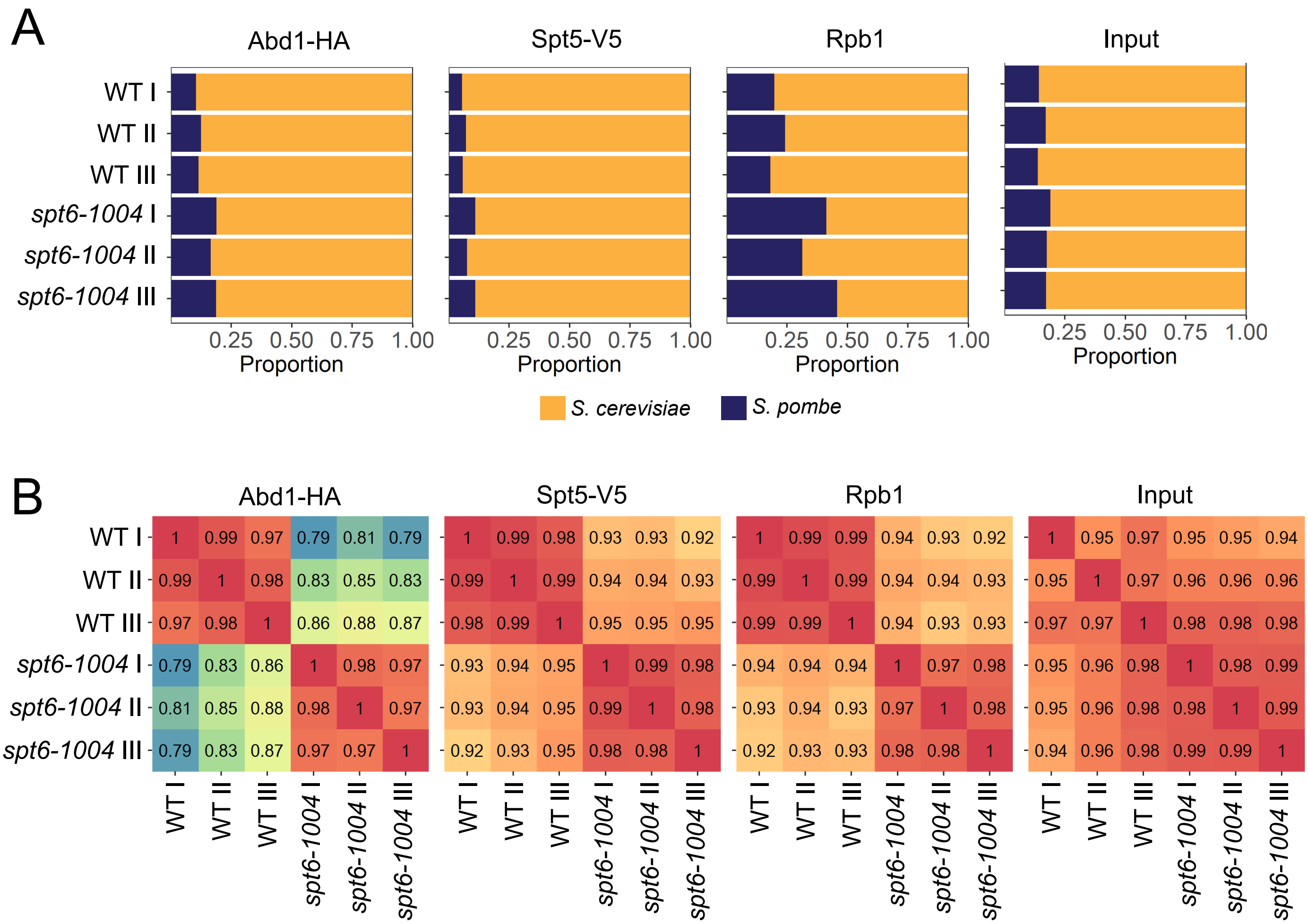

Supplementary Figure 2. Quality control analysis of ChIP-Seq data
(A) Bargraphs showing the proportion of reads in each library mapped to the *S. cerevisiae* or *S. pombe* (spike-in control) genome. (B) Correlation heatmaps for all 24 samples that were processed for ChIP-Seq. The numbers represent the Pearson correlation coefficient of coverage over 20 bp bins tiling the entire genome.

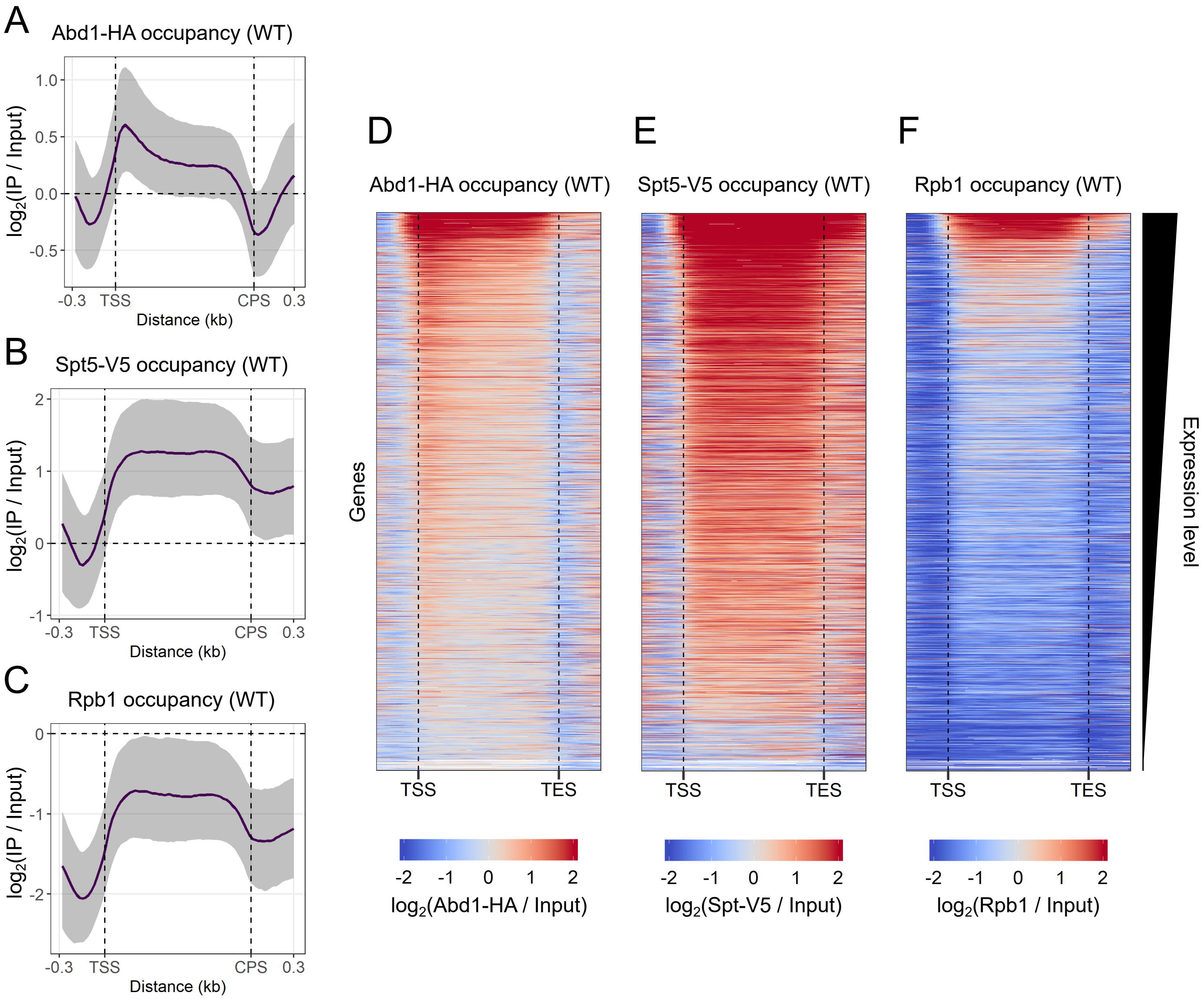

Supplementary Figure 3. Genome-wide recruitment of Abd1, Spt5 and RNAPII to transcribed regions in wild-type cells.
(A-C) Metagene plots of Abd1-HA (A), Spt5-V5 (B), and Rpb1 (C) occupancy normalized to input for 3522 non-overlapping protein-coding genes in wild-type cells. The purple trace indicates the median occupancy at a given position. The shaded area represents the interquartile range. (D-F) Heatmaps of Abd1-HA (D), Spt5-V5 (E), and Rpb1 (F) occupancy normalized to input for 3522 non-overlapping protein-coding genes in wild-type cells. All values that had a log fold change of below -2 or above 2 are set to -2 and 2 respectively. The genes are arranged in decreasing order of expression level as determined from RNA-Seq of wild-type cells (Reim *et al.*, 2020).

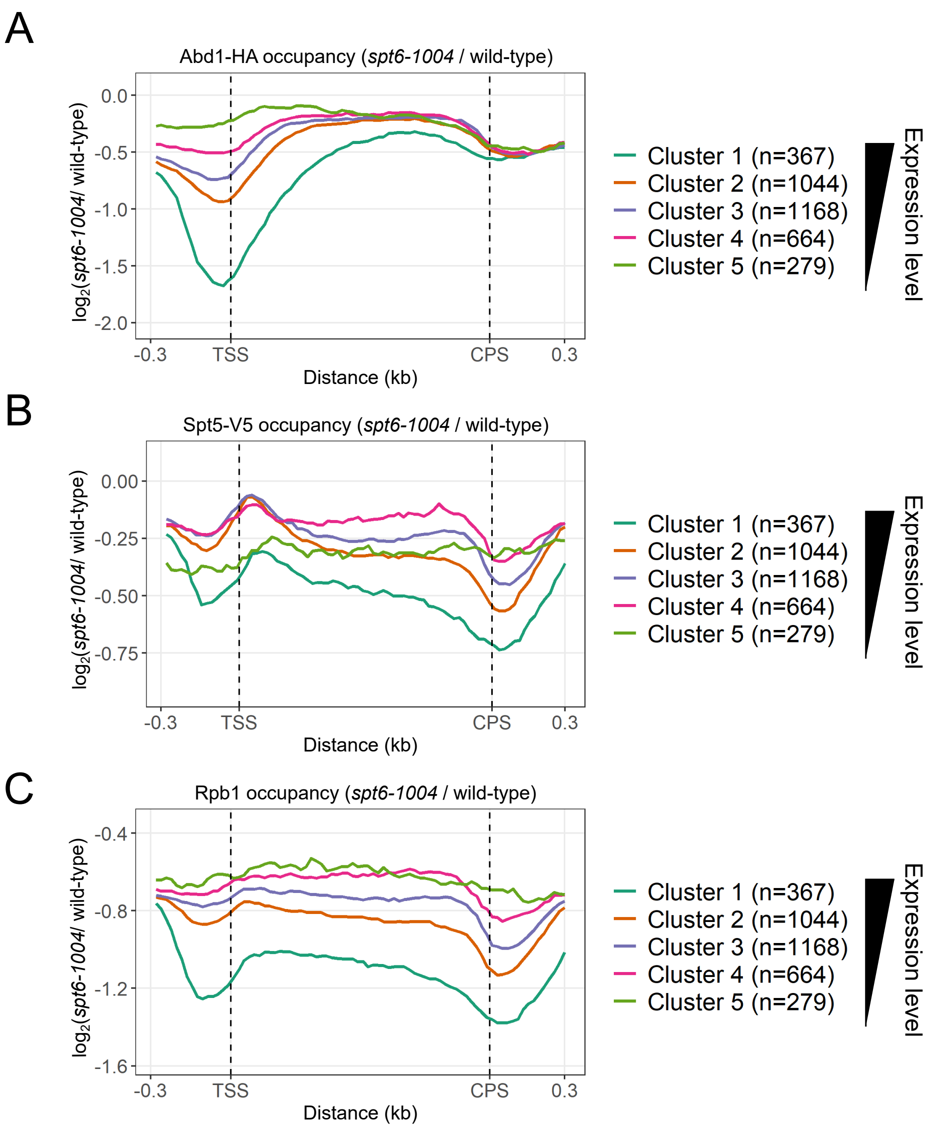

Supplementary Figure 4. Occupancy of Abd1, Spt5 and RNAPII over transcribed regions binned by their expression level in wild-type cells

Abd1-HA (A), Spt5-V5 (B) and Rpb1 (C) occupancy over 3522 non-overlapping protein coding genes binned by their expression levels in wild-type cells. The genes have been binned into five clusters using k-means clustering of the $\log_2$ normalized FPKM values from wild-type RNA-Seq data (Reim *et al.*, 2020). Each trace represents the median $\log_2$ fold change (*spt6-1004* / wild-type) in occupancy in a particular cluster.
